# Laparoscopic proctectomy for rectal cancer in an automated peritoneal dialysis patient: A case report

**DOI:** 10.1016/j.ijscr.2020.06.082

**Published:** 2020-06-24

**Authors:** Hao Li, Bin Wang, Linhua Jiang, Yizhou Yao, Xuchao Wang, Diyuan Zhou, Xinguo Zhu

**Affiliations:** Department of General Surgery, The First Affiliated Hospital of Soochow University, Suzhou, Jiangsu, China

**Keywords:** Peritoneal dialysis, Rectal cancer, Laparoscopic proctectomy

## Abstract

**Introduction:**

There is little evidence to support the safety of peritoneal dialysis (PD) in restarting immediately after abdominal surgery. It is also unclear whether early recovery of PD in minimally invasive abdominal surgery reduces the risk of complications. This is the first case report of laparoscopic proctectomy in a patient on PD.

**Presentation of case:**

A 66-year-old female patient with end-stage renal disease underwent daily automated peritoneal dialysis. Colonoscopy in the patient suggested rectal cancer. After laparoscopic rectum resection, restart PD on post-operative day 2. Two weeks after the operation, the patient's surgical wound healed well and renal function recovered to the same as before, and the original PD plan was maintained without obvious discomfort.

**Discussion:**

Multiple other case reports have shown that PD has been safely resumed after various operations. However, PD patients undergoing abdominal surgery may cause serious complications. Maybe the peritoneal dialysis restores immediately after surgery should consider some conditions.

**Conclusion:**

We think that it may be safe for PD patients to continue to maintain PD after laparoscopic rectum resection without the need for temporary hemodialysis transition. More cases will be collected in the future to consolidate this conclusion and provide reference for the diagnosis and treatment of similar cases in the future.

## Highlights

• This is the first case report of laparoscopic proctectomy in a patient on peritoneal dialysis (PD).
• It may be safe for PD patients to continue to maintain PD after laparoscopic rectum resection without the need for temporary hemodialysis transition.
• The patient’s surgical wound healed well and renal function recovered to the same as before.
• The peritoneal dialysis adjustment program is beneficial to patient.
• This case report reflects the treatment of patients in multiple disciplines together, and reflects the advantages of the multidisciplinary diagnosis and treatment model.

## 1 Introduction

Peritoneal dialysis is to use the peritoneum as a semi-permeable membrane, inject dialysate into the peritoneum, with the help of plasma in capillaries and peritoneal cavity dialysate in the solute gradient and osmotic gradient, through the principle of dispersion to remove metabolic waste and excess water in the body [1]. There is little evidence to support the safety of peritoneal dialysis (PD) in restarting immediately after abdominal surgery [2]. It is also unclear whether early recovery of PD in minimally invasive abdominal surgery reduces the risk of complications. In particular, there have been no reports on perioperative management of PD in patients undergoing Laparoscopic proctectomy for rectal cancer. This is the first case report of laparoscopic proctectomy in a patient on automated PD.

## 2 Presentation of case

**Fig. 1:**
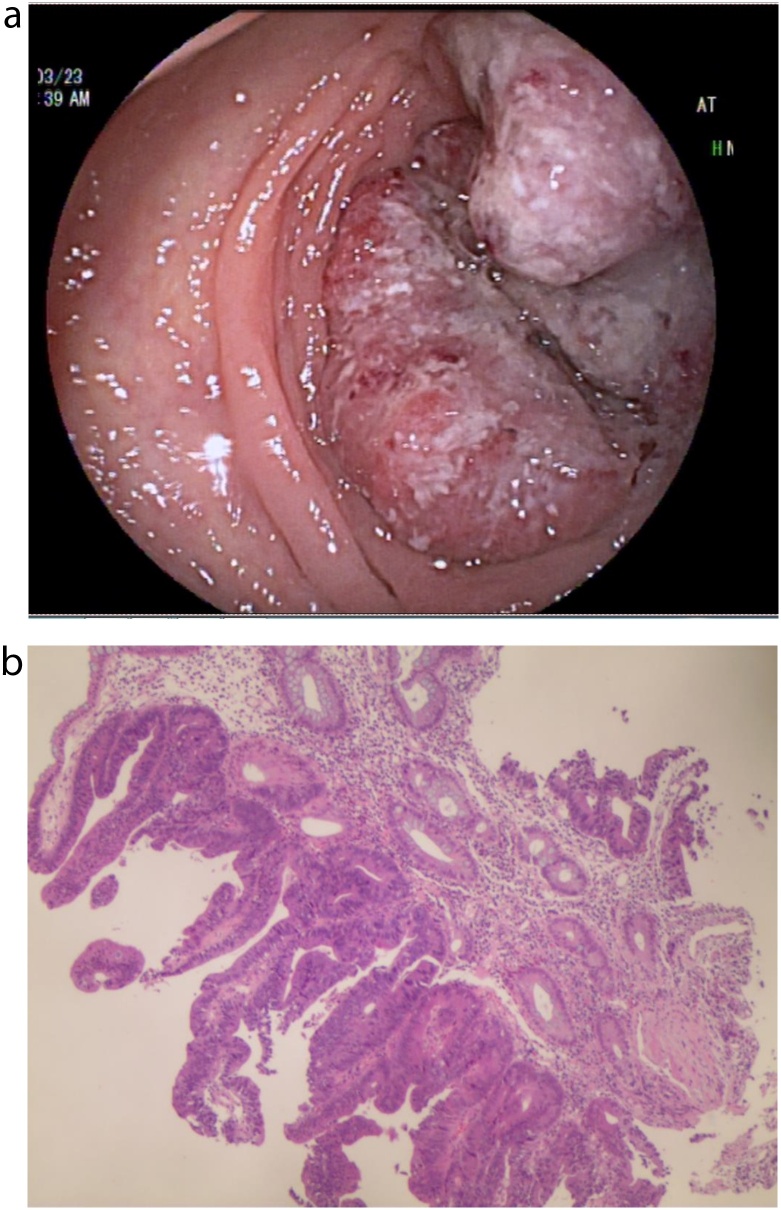
The colonoscopy view of the tumor (Fig. 1a) and the pathological section of the tumor (Fig. 1b).

**Fig. 2:**
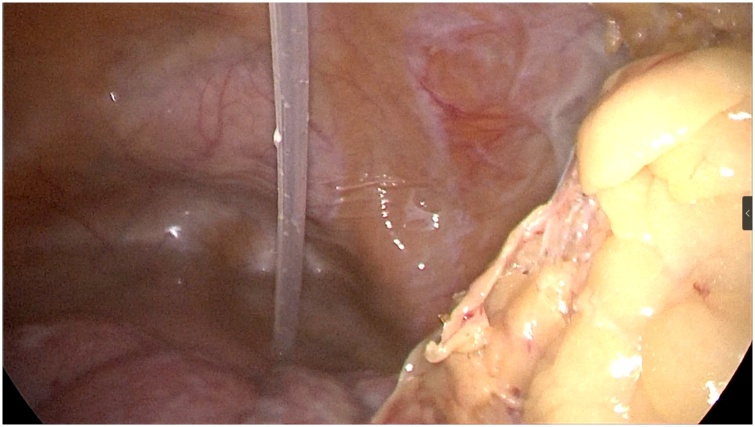
Intraoperative exploration of the field of vision, and the location of the peritoneal dialysis tube.

**Fig. 3:**
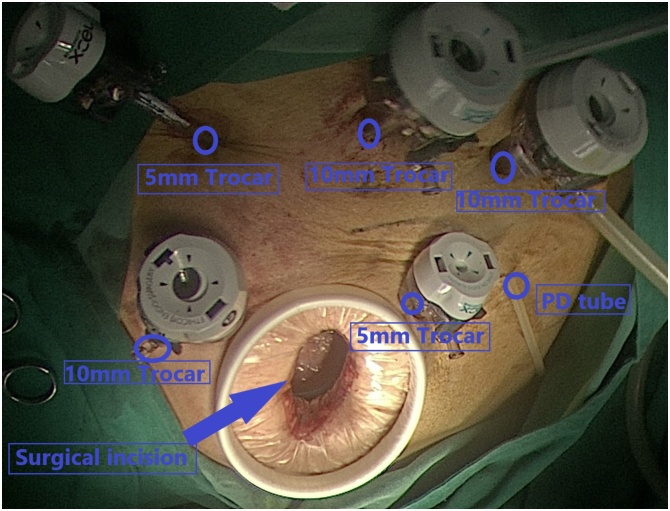
The position of the conventional surgery incision and trocar.

**Fig. 4:**
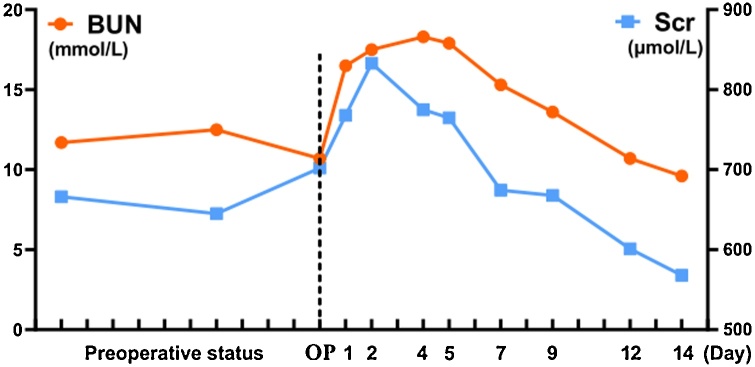
The changes of urea nitrogen and creatinine. OP means the operation day; the number in the abscissa is the day after the operation (1: the first day after the operation; 2: The second day after the operation, etc) ; BUN: urea nitrogen; Scr: creatinine.

A 66-year-old female patient with end-stage renal disease due to hypertensive nephropathy underwent daily automated peritoneal dialysis. Due to the change of bowel habits, colonoscopy and pathological biopsy were performed, and the diagnosis was rectal cancer (Fig. 1a–b). She has type 2 diabetes, high blood pressure, renal anemia, weekly subcutaneous injections of erythropoietin, and daily oral levamlodipine besylate to control blood pressure. The patient did not suffer from familial genetic disease and psychological disease. By referring to CT and MRI, The operation was determined as laparoscopic rectal resection and temporary ileostomy, peritoneal dialysis tube wasn't pulled out in the surgery, intraoperative probe (Fig. 2), and the position of the conventional surgery incision and trocar (Fig. 3). During the operation, a large amount of peritoneal fluid was found in the abdominal cavity. Local greater omentum adhesion was observed in the right lower abdomen. The tumor was found in the rectum about 3 cm above the peritoneum reflex, successfully completed the TME (total mesorectal excision) and ileum colostomy, a 5 cm incision was made in the middle of the lower abdomen, then the abdominal wall was opened, the tumor sample was taken out and the abdominal wall was sutured in layers, and a pelvic drainage tube was indwelled. 1.5 g cefuroxime sodium was given intraoperatively, and 3 g/day cefoperazone sulbactam sodium was given in the first three days after surgery to prevent infection. After consulting with the nephrologist and reviewing the literature, we communicated with the patient about the safety and related risks of direct peritoneal dialysis after surgery, the patient expressed her willingness to perform peritoneal dialysis first. Then we try to get patient to start PD on the second day after surgery, and the principle of PD is more frequencies and less doses. Peritoneal dialysis adjustment program is shown in (Table 1). We trained her family on post-op care, such as cleaning the PD tube and performing the PD according to the established protocol. In the meantime, there are professional nurses to take care of the patient. Renal function was regularly reviewed after surgery. The changes of urea nitrogen and creatinine are shown in (Fig. 4). The patient developed fever on the 9th day after surgery, the highest temperature is 38.6 °C, routine blood results: white blood cells 6.93*10^9^/L, neutrophil percentage 67.5%. The culture of abdominal drainage fluid indicated that escherichia coli was positive, but the patient didn't show signs of peritonitis, and then cefoperazone sulbactam sodium 3 g/day was given to the patient. Thereafter, the patient's temperature has been in the normal range, and the re-examination of abdominal drainage fluid showed no bacterial growth and there was no abnormality in blood routine. The patient was discharged from the hospital two weeks after surgery without surgical complications or PD related complications (such as dialysate leakage, wound infection or rupture). Patient compliance was good throughout the treatment, and we assess patient tolerance by monitoring blood routine, renal function, and vital signs. After one month of follow-up, no serious anastomotic leakage or peritonitis complications were found, and peritoneal dialysis was performed as usual without obvious discomfort [3].

**Table 1:** Peritoneal dialysis adjustment program.

Time point	preoperative	operation	POD 2	POD 3-10	POD 11-14	POD15 - Now
05:00				800 ml (1.5%)	1000 ml (1.5%)	
07: 00	2000 ml (1.5%)		800 ml (1.5%)			2000 ml (1.5%)
08: 00				800 ml (1.5%)	1000 ml (1.5%)	
10: 00	2000 ml (1.5%)		800 ml (1.5%)	800 ml (1.5%)	1000 ml (1.5%)	2000 ml (1.5%)
12: 00				800 ml (1.5%)		
15: 00	2000 ml (1.5%)		800 ml (1.5%)	800 ml (1.5%)	1000 ml (1.5%)	2000 ml (1.5%)
17: 00				800 ml (1.5%)		
20: 00	2000 ml (2.5%)		800 ml (2.5%)	800 ml (1.5%)	1000 ml (1.5%)	2000 ml (2.5%)
22: 00				800 ml (2.5%)	1000 ml (2.5%)	
**Total**	**8000** **ml**	**0** **ml**	**3200** **ml**	**6400** **ml**	**6000** **ml**	**8000** **ml**

This is the adjustment of the peritoneal dialysis solution at different time points every day. The following is an explanation of some nouns: Preoperative: preoperative program; POD 2: The second day after the operation; POD 3-10: Days 3 to 10 after surgery; 2000 ml (1.5%):2000 ml peritoneal dialysis solution at a concentration of 1.5%; 800 ml (2.5%):800 ml peritoneal dialysis solution at a concentration of 2.5%.

## 3 Discussion

Peritoneal dialysis (PD) is an effective treatment for patients with end-stage kidney disease. Compared with hemodialysis, PD therapy has several advantages, such as the maintenance of residual renal function and quality of life, mild dietary regulations, and low impact of cardiovascular dynamics [4]. Multiple other case reports have shown that PD has been safely resumed after various gastrointestinal operation, including appendectomy, laparoscopic cholecystectomy, elective open and laparoscopic hernia repair, and laparoscopic hemicolectomy [[5], [6], [7], [8], [9]]. However, PD patients undergoing abdominal surgery may cause serious complications, such as dialysis fluid leakage, wound infection, incisional hernia and so on [10]. Especially rectum resection destroyed the pelvic floor peritoneum, which has certain influence on the effect of PD, and the incidence of anastomotic fistula in rectal resection is higher than that of the colon, because the blood vessels around the anastomosis are easier to damage [11]. In addition, the peritoneal dialysate will change the intra-abdominal pressure and will leak out of the incision, thus affecting the wound healing [2]. The value of laparoscopic surgery in maintaining patients with peritoneal dialysis has been recognized [10,12]. Many reports indicate that the use of laparoscopy for PD patients with different pathological conditions is feasible, safe and successful [5,6,11]. The conventional wisdom is that PD patients should have their peritoneal catheter removed during rectal resection because of the higher risk of contamination and peritonitis [6]. In short, PD patients have more complications after undergoing rectal resection. Early recovery of peritoneal dialysis after surgery may lead to serious complications such as anastomotic leakage or intra-abdominal abscess.

In order to allow patient to recover peritoneal dialysis more quickly after surgery, we took relevant measures. First, the surgical plan chose laparotomy instead of open surgery. The smaller abdominal incision in laparoscopic surgery reduced the incidence of these complications, and postoperative risk of intestinal adhesions is reduced. Laparoscopic surgery is well accepted as a more conservative procedure compared with open surgery, with lower peritoneal membrane stress and better preservation of peritoneum integrity [11]. The patient has only a small incision in the abdomen, which is used to take out the removed tumor tissue. And we sutured the incision layer by layer and repeated flushing to reduce wound complications. Meanwhile, in order to avoid the occurrence of infectious peritonitis caused by anastomotic leakage and the loss of peritoneal dialysis function, we additionally performed a temporary ileostomy (Ileostomy will be re-received after a second operation in half a year). Prophylactic enterostomy has a protective effect on anastomotic leakage after rectal cancer surgery, can reduce local and systemic symptoms, promote the healing of anastomotic leakage, and reduce the reoperation rate and mortality [11]. For the purpose of reducing the impact of abdominal fluid on the wound, we enjoined the patient to keep lying down and reduced the amount of peritoneal dialysate per time after surgery, so that the contact between the incision and peritoneal dialysate is much less, and the direct or indirect effect of peritoneal dialysate on the incision is also minimized.

Of course, this situation is not suitable for every patient. For these cases, we recommend that the peritoneal dialysis be restored immediately after surgery to consider the following points: 1. The patient’s physical condition may not have a serious basic medical history; 2. The patient’s BMI should be at within a reasonable range, it should not be too fat, which will affect the surgical suture and wound healing; 3. The stage of the tumor should not be too late, and it should reach the point where it can be successfully removed by surgery; 4. Preventive anti-infection during the perioperative period, the day before the operation and the first 5 days after the operation, antibiotics should be used rationally to prevent infection, and the abdominal tube should be cleaned regularly; 5. Preventive stomas should be performed as much as possible to avoid anastomotic leakage; 6. Subtle manipulation and careful hemostasis should be done throughout the whole procedure in order to reduce the risk of remnant postoperative intra-abdominal fluids.

## 4 Conclusion

Laparoscopic proctectomy represent a valid and safe surgical intervention to treat rectal cancer [11], avoiding PD drop-out and favoring the chance of long-lasting PD patient’s survival. It may be safe for PD patients to continue to maintain PD after laparoscopic rectum resection without the need for temporary hemodialysis transition. Meanwhile, we adjusted the peritoneal dialysis program and found that the patient’s renal function recovered even better than before surgery. This case report fills the blank of whether peritoneal dialysis can be performed after surgery for rectal cancer patients, and provides a reference for the treatment of similar cases in the future. We have innovatively adjusted the peritoneal dialysis program to make the patient’s kidney function recover better, which may bring inspiration to the course and effect of peritoneal dialysis. In short, we feel that this case report has important significance in peritoneal dialysis and abdominal surgery. In this case, no complications were observed, and we suggest that the recommended regimen could be considered in this case in the hope of reducing complications and mortality in these patients.

## Declaration of Competing Interest

We declare that we do not have any commercial or associative interest that represents a conflict of interest in connection with the work submitted.

## Funding

No funding was received for this study.

Xinguo zhu is the study sponsor, he decided to submit the manuscript for publication; Hao Li wrote the manuscript; Bin Wang, Linhua Jiang and Yizhou Yao collected the date; Xuchao Wang and Diyuan Zhou analysed the data.

## Ethical approval

This case report has been published with the patient’s informed consent and has been reviewed by the ethics committee of the First Affiliated Hospital of Soochow University.

## Consent

We have obtained written and signed consent to publish a case report from the patient prior to submission. Patient’s name, initial, and hospital number have not been used.

## Author contribution

Xinguo zhu determined the research design;

Hao Li wrote the manuscript;

Bin Wang, Linhua Jiang and Yizhou Yao collected the date;

Xuchao Wang and Diyuan Zhou analysed the data.

## Registration of research studies

N/A.

## Guarantor

Xinguo Zhu is the guarantor.

## Provenance and peer review

Not commissioned, externally peer reviewed.
